# Optimising decision making on illness absenteeism due to fever and common infections within childcare centres: development of a multicomponent intervention and study protocol of a cluster randomised controlled trial

**DOI:** 10.1186/s12889-017-4602-3

**Published:** 2017-07-26

**Authors:** K. K. B. Peetoom, R. Crutzen, J. M. H. A. Bohnen, R. Verhoeven, H. J. M. G. Nelissen-Vrancken, B. Winkens, G. J. Dinant, J. W. L. Cals

**Affiliations:** 1 Care and Public Health Research Institute Department of Family Medicine, Maastricht University, Maastricht, The Netherlands; 2 Care and Public Health Research Institute, Department of Health Promotion, Maastricht University, Maastricht, The Netherlands; 3 Care and Public Health Research Institute, Department of Methodology and Statistics, Maastricht University, Maastricht, The Netherlands; 4 Dutch Institute for Rational Use of Medicine, P.O. Box 3089, 3502 GB Utrecht, The Netherlands

**Keywords:** Childcare centres, Fever, Common infections, Intervention mapping, Illness absenteeism

## Abstract

**Background:**

Evidence has shown that children 0-4 year-old attending childcare are prone to acquire infections compared to children cared for at home, with fever being the most common symptom. Illness absenteeism due to fever and common infections is substantial and mostly driven by unrealistic concerns and negative attitude towards fever of both childcare staff and parents, resulting in illness absenteeism from childcare, work absenteeism among parents and healthcare service use. The objective of this study is to optimise decision making among childcare staff on illness absenteeism due to fever and common infections in childcare. Underlying determinants of behavioural change were targeted by means of a multicomponent intervention.

**Methods:**

A multicomponent intervention was developed to improve decision making, using the stepwise approach of Intervention Mapping, and in close collaboration with stakeholders and experts. The intervention consisted of 1) a two-hour educational session on fever among childcare staff; 2) an online video for childcare staff and parents emphasising key information of the educational session; 3) a decision tool for childcare staff and parents in the format of a traffic light system to estimate the severity of illness and corresponding advices for childcare staff and parents; 4) an information booklet regarding childhood fever, common infections, and self-management strategies for childcare staff and parents.

The multicomponent intervention will be evaluated in a cluster randomised trial with a 12-week follow-up period and absenteeism due to illness (defined as the percentage of childcare days absent due to illness on the total of childcare days during a 12-week period) as primary outcome measure. Secondary outcome measures are: incidence rate and duration of illness episodes, knowledge, attitude, self-efficacy, and risk perception on fever and common infections of childcare staff and parents, healthcare service use in general and paracetamol use, and work absenteeism of parents.

**Discussion:**

This study aims to develop a multicomponent intervention and to evaluate to what extent illness absenteeism due to fever and common infections can be affected by implementing a multicomponent intervention addressing decision making and underlying determinants among childcare staff and parents of children attending daycare.

**Trial registration:**

NTR6402 (registered on 21-apr-2017).

**Electronic supplementary material:**

The online version of this article (doi:10.1186/s12889-017-4602-3) contains supplementary material, which is available to authorized users.

## Background

Infectious diseases, such as fever, are common in children 0-4 year-old. Especially children who attend childcare are more prone to acquire respiratory and gastro-intestinal infections compared to home-cared children, particularly during the age of 0-2 years [1–4]. This may be explained by the fact that their immunological and respiratory tract system is still developing when attending childcare. In addition, children in childcare encounter a higher amount of microbes and have more frequent and more close contacts with other children compared to home-cared children [1, 5–10].

Infectious diseases are a common reason for illness absenteeism in childcare, with children in childcare experiencing on average 6.6-11.4 illness episodes per year, constituting an average illness absenteeism from childcare of 22.9 days [4, 5, 11, 12]. Societal costs for infectious diseases are twice as high per illness episode in children attending childcare as non-attenders, constituting a total economic burden of €97 million per year in The Netherlands. This burden is mostly due to work absenteeism among parents, with an average of 1.5 work days lost per child’s illness episode [2].

The decision regarding illness absenteeism due to fever and common infections may be driven by both parents and childcare staff. Elevated temperature or fever can create a range of concerns among those caring for the child. At the core of these concerns is the misconception that the temperature elevation represents the severity of underlying illness, rather than a relatively benign symptom resulting from an infectious disease. In addition, harmful consequences of fever are feared and body temperature is measured several times per day to monitor disease progression [13–25]. The term fever phobia has been used to describe an exaggerated, unrealistic and unfounded fear of fever expressed by parents, healthcare professionals and childcare staff [24]. An important driver of fever phobia is a lack of knowledge regarding fever pathophysiology, but also previous (negative) experiences, beliefs, sense of personal control, perceived threat of illness, inconsistencies in healthcare providers’ advices, and the concern of not recognising a serious illness on time [14–16, 25–29]. In turn, fever phobia influences general practitioner (GP) consultations for childhood fever [5, 6, 22, 30–33]. Similarly, fever phobia is present in childcare staff and causes substantial illness absenteeism in children, work absenteeism among parents and healthcare service consultations by referring parents to the GP [2, 6, 13, 16, 17, 20, 33–37]. Despite the development of several educational interventions, fever phobia, misconceptions and unrealistic concerns are still present in parents, healthcare professionals and childcare staff [36, 38–40].

Educational interventions on fever and common infections targeting childcare staff are scarce and focus merely on hygiene prevention [12, 30, 41–43]. These hygiene prevention interventions are successful in reducing illness incidences rates, children’s illness absenteeism and parents’ work absenteeism [12, 30, 41, 42]. However, educational interventions for childcare staff regarding fever pathophysiology, infectious diseases and self-management strategies could be beneficial for diminishing fever phobia, since childcare staff strongly influence childcare illness absenteeism, work absenteeism for parents, and parental healthcare seeking behaviour [34].

As such, it is hypothesised that educating childcare staff on fever and infectious diseases may optimise decision making processes on illness absenteeism due to fever and common childhood infections, resulting in reduced childcare absenteeism, work absenteeism and use of healthcare services. To achieve this, it is important to obtain insight in which behavioural determinants influence decision making on illness absenteeism in childcare, and to target these determinants in the intervention. As childcare staff are also an important source of information for parents [33], it is expected that improved behavioural determinants of decision making among childcare staff will result in improved parental decision making on illness absenteeism.

This paper describes the development of a multicomponent intervention aiming to optimise decision making among childcare staff on illness absenteeism due to fever and common infections in 0-4 year-old attending childcare, and describes the study protocol of the cluster randomised trial evaluating the developed multicomponent intervention in childcare.

## Methods

This section describes the development of a multicomponent intervention, based on the Intervention Mapping approach (IM), a protocol for developing theory-based and evidence-based interventions [44]), followed by the study protocol of the cluster randomised trial (cluster RCT) for evaluating the multicomponent intervention in childcare. The study protocol is reported according the guidelines of Standard Protocol Items: Recommendations for Interventional Trials (SPIRIT) [45, 46].

### Development of multicomponent intervention

A multicomponent intervention was developed using IM with the aim to target underlying determinants of behavioural change of childcare staff in order to optimise their decision making with respect to illness absenteeism due to fever and common infections in childcare.

The first step of the development process was the assessment of extensive needs to explore difficulties towards childhood fever, causes, consequences and implications for practice by means of a systematic review, a survey study among parents of children visiting childcare, and three qualitative studies among general practitioners, well-child clinic professionals, and childcare staff [17, 25, 39]. Initial data analyses from the qualitative study among childcare staff showed that fever phobia was common and emanated from a lack of knowledge and education on fever and common childhood infections [unpublished observations]. Childcare staff expressed that their current knowledge was merely experience-based, both individually and as group sharing experiences, and guided by the operating protocol of their childcare organisation. An uncomfortable feeling was also expressed in case of an increase in the child’s body temperature, caused by the concern that an increased temperature would represent the severity of illness. This finding was similar to previous studies [15, 16, 18, 19, 24, 25]. In agreement with another study, data from the qualitative study demonstrated that body temperature is frequently measured to monitor illness progression gives a sense of control over the illness [14]. Like parents, childcare staff especially fear febrile seizures as a result of an elevating body temperature and irregular administration of paracetamol [16]. In response to their concerns, childcare staff prefer to exclude children with a fever from childcare and return the responsibility to the parents. In agreement with previous research [17, 34], parents are also actively advised by the childcare staff to consult the GP for a check-up.

These research data demonstrate that decision making on illness absenteeism due to fever and common infections in childcare is a behavioural outcome, which could be optimised in order to reduce childcare absence, healthcare service use, and work absenteeism. Based on data from the needs assessment, the development and evaluation of the intervention will focus on the following four behavioural determinants that are fundamental for the decision making process on illness absenteeism within childcare: knowledge, attitude, self-efficacy and risk perception. Knowledge on pathophysiology of fever, febrile seizures and self-management strategies such as paracetamol use is seen as prerequisite for changing attitude [47, 48]. Attitude concerns an individual’s evaluation of performing a behaviour by balancing pros and cons [47]. For example, childcare staff showed a negative attitude towards fever, resulting in concerns, cautious behaviour and the tendency to send febrile children home. Self-efficacy is defined as the belief in the ability to perform a certain behaviour in a specific situation [44]. For example, childcare staff showed to act cautiously when a child has a (high) fever as they feel insecure in advising parents on GP consultation or paracetamol use, because they are not medically educated and measure body temperature several times per day to monitor illness progression. Risk perception relates to the assessment of risks towards performing a certain behaviour. For example, childcare staff perceive fever and febrile seizures as harmful to the child’s health while fever and most febrile seizures are not harmful in itself. Additional file 1 presents an overview of all change objectives (i.e., what has to change at the determinant level) and performance objectives (i.e., sub-behaviours that are essential to achieve the desired behavioural outcome.

Subsequently, theory-based methods were selected and translated into practical applications to target all four behavioural determinants (Additional file 2) and resulted in a multicomponent intervention, including: 1) a two-hour educational session on fever among childcare staff; 2) an online video for childcare staff and parents emphasising key messages of the educational session; 3) a decision tool in the format of a traffic light system to estimate the severity of illness and corresponding advices by childcare staff and parents; 4) an information booklet regarding childhood fever and common infections for childcare staff and parents.

#### 1. Educational session

The two-hour educational session involved a group meeting with childcare staff from one or more childcare intervention centres. Three main topics were discussed: pathophysiology of fever, treatment of fever, and febrile seizures. These topics were based on data from the qualitative study among childcare staff and existing scientific literature [15, 40, 49, 50]. The session aimed to discuss existing and widely held misconceptions on childhood fever, as described in Additional file 1. The content provided in the educational session was based on the current guideline “Childhood Fever” of the Dutch College of General Practitioners (NHG) and the content of the interactive booklet on childhood fever [51, 52]. The educational session included quiz questions on pathophysiology of fever, febrile seizures and self-management strategies such as the correlation between the height of fever and illness’ severity, and was led by an experienced moderator with a background in pharmacy [HN] and a general practitioner was present to answer medical related questions. The principal investigator [KP] was present to provide more information on the project, and to introduce, explain and practice the use of the decision tool.

#### 2. Online video

Based on the information provided during the educational sessions, questions asked by childcare staff during the session and take home messages formulated by the childcare staff, an online video of 7 min was composed using written text, illustrations and animations to convey information. The aim of this video was to further educate participating staff about fever pathophysiology, treatment of fever, and decision making regarding childcare attendance. This video was sent to childcare staff and parents approximately 6 weeks after the educational session. The video served as a booster regarding the information discussed during the educational session.

#### 3. Decision tool

**Fig. 1:**
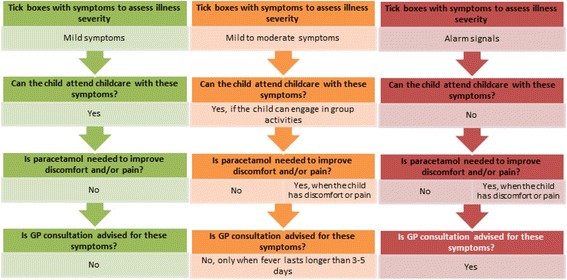
Schematic overview of the decision tool on illness severity and accompanying advices

The aim of the decision tool is to support childcare staff and parents in assessing the illness severity of the child’s symptoms, as well as to guide them towards accompanying advices regarding childcare attendance, and self-management strategies (paracetamol use and GP consultation) in order to optimise their decision making on illness absenteeism from fever and common infections. The decision tool is based on the current guideline “Childhood Fever” of the Dutch College of General Practitioners (NHG) [52], Dutch Public Health Services [53], and the operation protocol on illness from the participating childcare organisation. The decision tool was designed based on the traffic light system developed by de Bont et al. [51] and aims to support with assessing the degree of severity of the child’s illness based on behavioural and physical symptoms of the child. The severity was categorised using the traffic light colours with “Green” representing mild and harmless symptoms, “orange” representing mild to moderate symptoms, and “red” representing alarm signals. Based on these colour categories, the decision making processes among childcare staff and parents could be optimised regarding childcare attendance, paracetamol use and GP consultations. Figure 1 presents a schematic overview of the decision tool.

#### 4. Interactive information booklet

The interactive information booklet is an existing intervention, which was developed using a multistage process and evaluated in a large cluster randomised controlled trial in 20 GP out-of-hours services in The Netherlands between November 2015 - April 2016 [51]. The booklet contains a traffic light system for childhood fever in combination with other infectious symptoms, which provides advice on whether to consult a GP (red) or use self-management strategies (green, orange) for childhood fever in general. Furthermore, it provides information on the benefits and harms of antibiotic treatment, an overview of natural duration of common infections in children, and it presents a table with weight-banded paracetamol dosage schemes. The content of the interactive information booklet is in line with current guidelines of the Dutch College of General Practitioners (NHG) [52].

A pilot study was conducted in order to evaluate the individual components of the intervention in an independent childcare organisation in the region of South-Limburg in November 2016, which will not participate in the trial. This childcare organisation consists of six childcare centres, five pre-school playgroups, and eight school care facilities. According to this pilot study, the order of the educational session was changed and the text and lay-out of the decision tool, survey and the registration form were edited. Furthermore, the decision tool was pretested to several end-users of the participating childcare organisation in order to verify whether the method, content and lay-out matched the requirements of end-users.

### Cluster randomised controlled trial

#### Study setting

Approximately 45% of 0-4 year-old children attend any form of childcare in The Netherlands [54], of which 79% attend childcare provided by commercial organisations. In such centres, educated staff care for, and contribute to the development and upbringing of children, until they progress to primary school [55]. Other forms of childcare in The Netherlands are host parents (16.5%), or the combination of commercial childcare and host parents (4.8%) [54]. This trial will be conducted in a large commercial childcare organisation in the Southern part of province Limburg, The Netherlands (Meerwaarde in ‘n Kinderleven (MIK) childcare organisation, Maastricht, The Netherlands). This organisation provides childcare to approximately 1380 children aged 0-4 years, distributed over 21 childcare centres. In addition, they facilitate pre-school playgroups and school care facilities. This part of The Netherlands has a wide variety of social economic status (SES), with more parents with a lower SES living in the larger cities [56].

### Participants

From the 21 childcare centres eligible for this study, 18 centres agreed to participate. The site managers of the participating childcare centres will be informed about the study content by means of written information and verbal elucidation. Childcare staff and parents with children at the participating childcare centres will be informed about the study content by means of a written information letter via email. All childcare staff with permanent tenure, fixed replacements and trainees working at the participating childcare centres are eligible to participate in the educational session. The other individual components of the intervention will be made available to all eligible childcare staff at intervention centres regardless of their employment and to parents of children attending the participating childcare centres. All children attending the participating childcare centres and their parents are eligible to participate in this study. It should be noted that children with chronic medical conditions will not be excluded from participation, because the focus of this study is on illness absenteeism from common infectious diseases among 0-4 year-old children, which also occur in children with chronic diseases. Children are excluded from the illness registration when they reach the age of 4 years and leave childcare to progress onto preschool.

### Outcomes

The primary outcome is absenteeism due to illness, defined as the percentage of days that the child is absent from childcare due to illness from the total number of childcare days during a 12-week period. This primary outcome will be used to evaluate the potential behavioural change in decision making on illness absenteeism, resulting from the multicomponent intervention that was developed to target behavioural determinants such as knowledge, attitude, self-efficacy and risk perception.

Secondary outcome measures are: 1) knowledge, attitude, self-efficacy and risk perception towards fever and common infections are measured among childcare staff and parents; 2) incidence rate of fever and common childhood infections; 3) number and duration of illness episodes in children who are called in ill or sent home because of illness during the study period, and whose parents complete a survey on this illness episode; 4) work absenteeism among parents who complete a survey when their child is called in ill or sent home because of illness during the study period; 5) healthcare service use for fever and/or common infections in children who are called in ill or sent home because of illness during the study period and of which parents complete an additional survey on this illness episode, 6) paracetamol use for fever and/or common infections in children who are called in ill or sent home because of illness during the study period and whose parents complete an additional survey on this illness episode.

Secondary outcomes will be measured using two different surveys on different time points and for a different target group (Table 1). Survey 1 measures number and duration of illness episodes (3), work absenteeism (4), healthcare service (5), and paracetamol. Survey 1 is only sent to parents of which the child was ill during the study period and provided informed consent. The content of this survey is in line with surveys used in previous studies [19, 34, 42, 57, 58]. Survey 2 measures knowledge, attitude, self-efficacy and risk perception among childcare staff, whereas survey 3 measures these behavioural determinants among parents. Three vignettes were included to evaluate the decision making process of childcare staff and/or parents in three different cases of ill children based on the traffic light system that was implemented in the decision tool. These surveys on knowledge, attitude, self-efficacy, and risk perception for childcare staff and parents are developed based on previous research [19, 34, 58–61]. Survey items were operationalised according theories’ definitions of behavioural determinants. Subsequently, survey items were piloted among childcare staff to check understandibility and interpretation of items [62].

**Table 1:** Time schedule of enrolment, intervention allocation, outcome assessments

			Study period						
	Enrolment	Allocation	Post-allocation						Close-out
Timepoint	*0*	0	*T*_*1*_	*T*_*2*_	*T*_*3*_	*T*_*4*_	*T*_*5*_	*T*_*6*_	
Enrolment:	X								
Eligibility screen	X								
Informed consent Survey 1 following child’s illness episode	X	X	X	X	X	X	X	X	
Informed consent Survey 2 knowledge, attitude, self-efficacy, risk perception childcare staff	X								
Informed consent Survey 3 knowledge, attitude, self-efficacy, risk perception parents									X
Allocation		X							
Interventions:									
Educational session Childcare staff		X							
Use of decision tool + booklet Childcare staff and parents		X	X	X	X	X	X	X	
Online video Childcare staff and parents					X				
Assessments:									
Children’s illness registration		X	X	X	X	X	X	X	
Survey 1 following child’s illness episode		X	X	X	X	X	X	X	
Survey 2 Knowledge, attitude, self-efficacy, risk perception Childcare staff	X								X
Survey 3 Knowledge, attitude, self-efficacy, risk perception parents									X

### Sample size

Our primary outcome is absenteeism due to illness, defined as the percentage of childcare days absent due to illness on the total of childcare days during a 12-week period. We performed a pilot study among parents with children attending 21 childcare centres of MIK in Spring 2016 to calculate illness absenteeism. For this pilot study, an average 4-week illness absenteeism of 9.9% (sd = 18.035) was reported [unpublished observations], which was in agreement with the absenteeism rate reported by Hedin et al. in Swedish children [63]. To calculate our sample size we assumed an illness absenteeism of 9.9% in the control group. This cluster RCT aims to achieve a clinically significant reduction of illness absenteeism due to fever and common infections by 4.5% in the intervention group [4, 30, 64]. To obtain this difference with 80% power, assuming a within group SD of 18.035 and a significance level α of 0.05, 253 participants per group (506 in total) are required (PS Power and Sample size program, independent-samples t-test). Due to cluster (childcare location) randomisation, where we assume a mean number of 55 children per cluster and an intra-cluster coefficient of 0.01 into account, based on our pilot and previous literature [65], this number increases to 390 participants per group. After correction for 10% loss to follow-up and 10% efficiency loss based on unequal cluster sizes [66], the required sample size is 482 participants per group (total *n* = 964). To meet this sample size, this study aims to recruit 18 clusters (childcare location) with 55 participating children per cluster, resulting in 990 children.

### Allocation

Stratified block randomisation is conducted at the level of childcare centres, with nine centres randomised to our multicomponent intervention and nine centres randomised to care-as-usual (control). Intervention centres will receive all four components of the multicomponent intervention, control centres will provide care as usual. Stratification is based on size of childcare centres regarding number of children attending a childcare center. A small childcare center is defined as having fewer than 65 children attending childcare, while a large childcare center is defined as having more than 65 children attending childcare. The randomisation procedure is conducted by an independent researcher via http://www.randomization.com/. Childcare staff and parents will not be blinded after intervention allocation due to the nature of the multicomponent intervention. However, they are blinded to the primary outcome measure. Researchers will be blinded during data analysis to the allocation status of childcare centres.

### Data collection

See Table 1 for an overview of enrolment, intervention allocation, and outcome measurements.

#### Primary outcome measure

A standardised paper-based illness registration form is developed in close collaboration with the participating childcare organisation (see Additional file 3). Six booklets each comprising 2 weeks of childcare will be provided to all participating childcare groups for the study period of 12 weeks. Childcare staff will register childcare attendance, childcare absenteeism because of illness, and absenteeism due to other reasons (e.g. holiday) on a daily basis. When children are called in ill or sent home ill, childcare staff will register initials of the child, infection symptoms and how many days the child is (partially) absent due to illness. In addition, childcare staff in the intervention group will also record decision tool use. Childcare staff in both groups will receive training and feedback on registration during a 2-week run-in period before the actual start of the trial (see Table 1). This method of illness registration is comparable to other studies [63, 67, 68].

#### Survey 1 following child’s illness episode

Parents will receive an electronic survey when their child was called in ill or is sent home from childcare because of illness when parents provide consent to receive this additional survey during the 12-week study period. Parents will receive this survey within 14 days after each time their child becomes ill, followed by a total of two reminders which are sent out every 3 days.

#### Survey 2 and 3 on knowledge, attitude, self-efficacy, and risk perception

Knowledge, attitude, self-efficacy and risk perception towards fever will be measured by means of 7-point likert scales in a survey that is distributed electronically and on paper. Childcare staff in intervention and control group will receive this survey at baseline (T0) and after the 12-week study period (T6) to measure the change in behavioural determinants between and within intervention and control group. Parents will receive the survey at T6 to measure the difference in behavioural determinants between intervention and control group.

### Analysis

Statistical analysis will be performed with SPSS version 21.0 and a two-sided *p*-value smaller than or equal to 0.05 will be considered statistically significant. Due to repeated measures (every 2 weeks for 12-weeks study period) and possible missing data, the primary outcome measure will be analysed using linear mixed models, where the covariance structure of the repeated measures will be chosen based on the lowest Bayes information criterion (BIC). Illness absenteeism will be computed every 2 weeks of registration for each location to take into account fixed closing days of the childcare center during public holidays. Missing outcome data will not be imputed, since a likelihood based approach will be used with linear mixed models. No missingness in the explanatory variables such as group (intervention or control), time (0, 2, …, 12 weeks), group*time, and stratification variable cluster size (large or small) is expected.

Secondary outcome measures will be analysed on different levels and in different ways. The survey on knowledge, attitude and practices for childcare staff will be analysed on three levels. Time is the lowest level (level 1), which is nested within staff members (level 2), which are again nested within location (level 3). Linear mixed models will be used for numerical outcomes and logistic mixed models or generalized estimating equations (GEE) for categorical outcomes, where an unstructured covariance structure will be used in case of 2 or three repeated measures, otherwise the covariance structure of the repeated measures will be chosen based on the lowest BIC. In addition, a random intercept on location level will be included to account for the nesting of staff members within a location. The same survey will be analysed for parents on two levels with parents as lowest level (level 1), which is nested in location (level 2), because only a post-allocation measurement will be performed to compare parents in intervention group with those in control group. In this case, a random intercept on location level is included in the model to account for the nesting of parents within a location. Survey outcomes are measured on 7-point likert scales and the average outcome (sd) will be calculated per item after assessing scale quality. The parent survey following child’s illness absenteeism will be analysed by using descriptive statistics. All data will be obtained, managed and monitored according to the guidelines of Good Clinical Practice.

## Discussion

This paper describes the development and study protocol for the evaluation of a multicomponent intervention in a cluster randomised trial that aims to improve decision making among childcare staff on illness absenteeism due to fever and common infections in childcare. The Intervention Mapping approach was used to systematically develop the intervention, according to the needs requirements of the end-users. A multicomponent intervention is developed to target the different behavioural determinants of decision making in several ways. This approach incorporated an extensive needs assessment based on results of own previous research and the available evidence from the scientific literature. The development included further a close collaboration with experts and end-users, and was pilot tested in a childcare organisation that will not take part in the main trial.

The content of the intervention is consistent with current guidelines of the Dutch College of General Practitioners, Dutch Public Health Services, and the operating protocol on illness from the participating childcare organisation. The multicomponent intervention will be evaluated in a cluster randomised controlled trial with nine childcare centres receiving the intervention and nine provide care-as-usual. There is a risk of contamination since childcare replacement staff may work in both intervention and control centres. Yet, we think this risk is minimal as the vast majority of staff works in one center only. Furthermore, to minimise this risk only permanent staff and fixed replacements will be allowed to attend the educational session. We chose to plan daily registration of absenteeism by childcare staff to minimise recall bias. Recall bias for secondary outcomes reported by parents will be minimised by sending out the survey within 2 weeks of illness of their child, followed by reminders every 3 days. Childcare staff will be blinded to the primary outcome measure to avoid bias.

Education on fever pathophysiology, febrile seizures, and self-management strategies is not structurally part of the training opportunities in Dutch childcare centres. This multicomponent intervention is in particular developed to educate childcare staff and parents on fever pathophysiology and self-management strategies by providing more background information and a newly developed decision tool. It was anticipated that optimising (determinants of) decision making among childcare staff and parents on illness absenteeism due to fever and common infections in childcare, may lead to substantial reductions in absenteeism from childcare, work absenteeism from parents, and high healthcare service use.

## Additional files

Additional file 1: Performance objectives regarding four behavioral determinants in decision making on illness absenteeism: knowledge, attitude, self-efficacy and risk perception. (DOCX 23 kb)

Additional file 2: Theory-based methods to target four behavioral determinants in decision making on illness absenteeism: knowledge, attitude, self-efficacy and risk perception. (DOCX 21 kb)

Additional file 3: Format of registration booklet on illness absenteeism. (DOCX 24 kb)

## Abbreviations

GP: General practitioner
IM: Intervention mapping
MIK: Meerwaarde in kinderleven
NHG: Dutch College of general practitioners
