# Supplementary material for: Optimising decision making on illness absenteeism due to fever and common infections within childcare centres: development of a multicomponent intervention and study protocol of a cluster randomised controlled trial
Source: BMC Public Health. 2017 Jul 26;18:61. doi: 10.1186/s12889-017-4602-3 (PMC5530501; doi:10.1186/s12889-017-4602-3)
Supplement: Supplementary file 1 — Performance objectives regarding four behavioral determinants in decision making on illness absenteeism: knowledge, attitude, self-efficacy and risk perception. (DOCX 23 kb) [file 12889_2017_4602_MOESM1_ESM.docx]

Appendix 1: performance objectives regarding our four behavioral determinants: knowledge, attitude, self-efficacy and risk perception.

| **Performance objective** | **Behavioural determinants** |  |  |  |
| --- | --- | --- | --- | --- |
|  | **Knowledge** | **Attitude** | **Self-efficacy** | **Risk perception** |
| P1: CC staff assess child’s illness severity on the combination of behaviour and other physical symptoms instead of (height of) fever only . | K1: Indicate that behaviour and other physical symptoms are indicators of illness severity, not (height of) fever. K2: Indicate that fever is a symptom of illness, not an illness itself.  K3: Indicate that fever alone is not an alarm signal. | A1: Acknowledge that height of body temperature/fever is not an indicator of illness’ severity. A2: Acknowledge behaviour and other physical symptoms than fever as most important indicators of illness’ severity. A3: Acknowledge that fever is beneficial and not harmful. | SE1: Express confidence in assessing child’s illness severity based on behaviour and other physical symptoms than fever. | RP1: Acknowledge that illness’ severity should be based on behaviour and physical symptoms other than fever only. RP2: Acknowledge that fever is not a single alarm signal/ indicator of illness’ severity. |
| P2: CC staff measure body temperature/fever not more than 1 time per day. | K4: Indicate that body temperature varies over the day and increases towards evening. K5: Indicate that monitoring body temperature is not an indicator of illness progression. K6: Indicate that fever is defined as 38°C or higher. | A4: Acknowledge that it is not necessary to measure body temperature regularly to monitor illness progression.  A5: Acknowledge that height of body temperature is not a single important indicator of illness’ severity. | SE2: Express confidence in measuring body temperature only once per day. SE3: Express confidence in assessing illness progression based on behaviour and physical symptoms than measuring body temperature. SE4: Express confidence in defining fever correctly. | RP3: Acknowledge that monitoring behaviour and other physical symptoms as a better assessment of illness progression instead of measuring body temperature >1 time per day. |
| P3: CC staff (advise parents to) only exclude (1)‘’red’’ children and (2)‘’orange’’ children not engaging in group activities. | K7: Indicate the difference between mild, moderate and alarm symptoms and when to exclude from childcare in relation to level of severity. K8: Indicate that behaviour and other physical symptoms are more important indicators of childcare attendance than fever (height). K9: Indicate that fever alone is not a reason to exclude children. | A6: Acknowledge that children with mild symptoms (green) and who are engaging in group activities (orange) can attend childcare. A7: Acknowledge that fever is not the most important decision rule toward attending childcare A8: Believe fever is not an alarm signal. | SE5: Express confidence in assessing when to exclude a child based on behaviour and other physical symptoms than fever. SE6: Express confidence in advising parents on childcare attendance. SE7: Express confidence when a child is ill at childcare with green/orange symptoms. | RP2: Acknowledge that fever is not a single alarm signal/ indicator of illness’ severity.  RP4: Recognize alarm signals (red). RP5: Recognize when to exclude ill children (red/orange) RP6: Recognize when exclusion is not needed(green/orange). RP7: Acknowledge that fever is not the reason to exclude. |
| P4: CC staff advise parents to consult the GP for ''red'' or ''orange'' symptoms with a fever longer than 3-5 days. | K10: Indicate that GP consultation is advised for alarm signals (red). K11: Indicate that GP consultation is advisable when a child has orange symptoms and fever duration is 3-5 days. | A9: Acknowledge parents do not have to be referred to the GP for mild to moderate symptoms. A10: Acknowledge parents have to be referred to the GP for consultation on alarm signals and when child’s fever lasts longer than 3-5 days in combination with orange symptoms. | SE8: Express confidence in assessing illness’ severity and when to consult a GP. SE9: Express confidence in advising parents on GP consultation. | RP8: Recognize to (advise to) consult a GP for alarm signals (red). RP9: Recognize when to consult a GP for orange symptoms. |
| P5: CC staff provide/advise about providing paracetamol for discomfort or pain and not to lower fever, and to base paracetamol dosing on child’s weight. | K11: Indicate that paracetamol may reduce discomfort and pain K12: Indicate that providing paracetamol to lower fever as single symptom is not beneficial and needed. K13: Indicate that GPs can still assess child’s illness severity when a child received a paracetamol. K14: Indicate that paracetamol dosing should be based on child’s weight. | A11: Acknowledge that paracetamol can reduce discomfort and pain and fever should not be lowered with paracetamol.  A12: Acknowledge that weight is the best indicator for paracetamol dosing. | SE10: Express confidence regarding when to provide paracetamol at childcare centers. SE11: Express confidence in advising parents on when to provide paracetamol. SE12: Express confidence in (advising on) paracetamol dosing. | RP10: Acknowledge that (a high) fever is not a single symptom that needs to be lowered with paracetamol. |
| P6: CC staff act less anxious when a child has a fever. | K15: Indicate that fever itself is not harmful.  K16: Indicate that chance on febrile seizure is rare. K17: Indicate that chance on febrile seizure is the highest when fever starts to rise. K18: Indicate that chance on febrile seizure is rare when fever is already high. K19: Indicate that most febrile seizures do not cause harmful effects. | A13: Acknowledge that febrile seizures are rare, not harmful in itself and mostly not occur when fever is already high. | SE13: Express confidence in coping with (future) febrile seizures. | RP11: Acknowledge that chance on developing febrile seizures is rare. RP12: Acknowledge that chance on febrile seizure is highest when fever starts to rise. RP13: Acknowledge that febrile seizure mostly do not occur when fever is already high.  RP14: Acknowledge that most febrile seizures do not cause harmful effects. |
