# Supplementary material for: Optimising decision making on illness absenteeism due to fever and common infections within childcare centres: development of a multicomponent intervention and study protocol of a cluster randomised controlled trial
Source: BMC Public Health. 2017 Jul 26;18:61. doi: 10.1186/s12889-017-4602-3 (PMC5530501; doi:10.1186/s12889-017-4602-3)
Supplement: Supplementary file 2 — Theory-based methods to target four behavioral determinants in decision making on illness absenteeism: knowledge, attitude, self-efficacy and risk perception. (DOCX 21 kb) [file 12889_2017_4602_MOESM2_ESM.docx]

Appendix 2: theory-based methods to target behavioral determinants

| **Determinant** | **Change objective** | **Theory-based method** | **Practical application in multicomponent intervention** |
| --- | --- | --- | --- |
| Knowledge | K1-K19 | Active learning | Posing quiz questions on common beliefs and facilitating discussion on answers during the educational session to promote central information processing. |
|  | K1-K19 | Discussion | Facilitating an open discussion on common beliefs during the educational session by means of a quiz. |
|  | K1-K19 | Advance Organisers | Poster with an explanation of the decision tool. Decision tool to assess illness’ severity based on behavior and physical symptoms. Information booklet on fever, common infections, and self-management strategies. |
|  | K1-K19 | Tailoring | The content of the educational session and film clip was tailored to the common beliefs/knowledge among childcare staff. The content of the information booklet was tailored to common questions on fever, infections, self-management strategies. The decision tool was tailored to the needs of childcare staff towards decision making on childhood fever and common infections (severity, childcare attendance, paracetamol use, GP consultation). |
|  | K1-K19 | Individualisation | In the educational session it is possible to pose individual questions on fever, common infections, self-management strategies and exclusion of ill children. |
| Attitude | A1-A13 | Belief selection | The content of the educational session and film clip was tailored to the common false beliefs among childcare staff. |
|  | A1-A13 | Persuasive communication | Participants are guided to change common false beliefs regarding fever by means of arguments to achieve a more positive attitude towards childhood fever in childcare. |
|  | A1-A13 | Active learning | Posing quiz questions on common beliefs and facilitating discussion on answers during the educational session to promote central information processing. |
|  | A1-A13 | Tailoring | The content of the educational session and film clip was tailored to the common beliefs among childcare staff.  The content of the information booklet was tailored to common questions on fever, infections, self-management strategies. The decision tool is tailored to the needs of childcare staff towards decision making on childhood fever and common infections (severity, childcare attendance, paracetamol use, GP consultation). |
|  | A1-A13 | Individualisation | In the educational session it is possible to pose individual questions on fever, common infections, self-management strategies and exclusion of ill children. |
|  | A1-A13 | Arguments | Arguments are presented in a persuasive way to weaken false beliefs during the educational session. In the film clip are key arguments from the educational sessions emphasized. |
| Self-efficacy | SE1-SE13 | Enactive mastery experiences | Each educational session starts with bringing in examples of ill children in childcare and how childcare staff acted upon. After introducing correct knowledge on fever and the decision tool in the session, participants apply the decision tool on the examples and receive feedback. |
|  | SE1-SE13 | Planning coping responses | Educational session and online film clip how to decide on illness severity, illness absenteeism, paracetamol use, GP advice based on the decision tool.  Educational session and online film clip discuss which steps to take when a febrile seizure occurs. |
|  | SE1, SE5-SE12 | Cue altering | Posters at childcare centers to attract attention of childcare staff and parents to use decision tool. |
|  | SE1, SE5-SE13 | Guided practice | Childcare staff are introduced to the decision tool during the educational session and practise during the session with own examples regarding decision making on illness absenteeism in childcare and receive feedback.  Educational session and online film clip discuss which steps to take when a febrile seizure occurs. |
|  | SE1-SE12 | Verbal persuasion | Communication strategy used during educational sessions. |
|  | SE1-SE12 | Facilitation | Providing decision tool to childcare staff and parents. Each childcare group receives an A3 memo pad with on each page a new decision tool to be filled in and which can be taken out. Posters to attract attention to use decision tool. Providing information booklet to all childcare staff and parents. |
| Risk perception | RP1-RP14 | Scenario-based risk information | Providing information on risks of harmful effects of fever/febrile seizures in educational session, film clip, information booklet. |
|  | RP1-RP14 | Conscious raising | Providing information and feedback on (questions related to) causes, consequences of fever/febrile seizures in educational session, film clip, information booklet. |
