# Supplementary material for: Optimising decision making on illness absenteeism due to fever and common infections within childcare centres: development of a multicomponent intervention and study protocol of a cluster randomised controlled trial
Source: BMC Public Health. 2017 Jul 26;18:61. doi: 10.1186/s12889-017-4602-3 (PMC5530501; doi:10.1186/s12889-017-4602-3)
Supplement: Supplementary file 3 — Format of registration booklet on illness absenteeism. (DOCX 24 kb) [file 12889_2017_4602_MOESM3_ESM.docx]

Appendix 3: format registration booklet illness absenteeism

| **Date: dd-mm-yyyy childcare group number: First name childcare staff:** |
| --- |
| Number of children who attend childcare for 2 dayparts today Number: |
| Number of children who attend childcare for 1 daypart today Number: |
| Number of children absent because of another reason than illness Number: |
| Total Dayparts missed: |
|  |
| **Children who were called in sick** |
| How many children were called in ill today? Number: |
| Initials child 1: Initials child 2: Initials child 3: Initials child 4: |
| Most important symptom (see table) |
| Child 1: Child 2: Child 3: Child 4: |
| How many dayparts were missed due to illness? |
| Child 1: Child 2: Child 3: Child 4: |
| Did you use (together with parent) the decision tool to take a decision? |
| Child 1: Child 2: Child 3: Child 4: |
|  |
| **Children who felt ill at childcare** |
| How many children felt ill today at childcare? Number: |
| Initials child 1: Initials child 2: Initials child 3: Initials child 4: |
| Most important symptom (see table) |
| Child 1: Child 2: Child 3: Child 4: |
| Did you have contact with the parent do decide on a plan of action? |
| Child 1: yes/no Child 2: yes/no Child 3: yes/no Child 4: yes/no |
| Did you use (together with parent) the decision tool to take a decision? |
| Child 1: yes/no Child 2: yes/no Child 3: yes/no Child 4: yes/no |
| Did you provide paracetamol to the child? |
| Child 1: yes/no Child 2: yes/no Child 3: yes/no Child 4: yes/no |
|  |
| **Children who were sent home due to illness** |
| How many children were sent home because of illness? Number: |
| Initials child 1: Initials child 2: Initials child 3: Initials child 4: |
| Most important symptom (see table) |
| Child 1: Child 2: Child 3: Child 4: |
| Did you have contact with the parent do decide on a plan of action? |
| Child 1: yes/no Child 2: yes/no Child 3: yes/no Child 4: yes/no |
| Did you use (together with parent) the decision tool to take a decision? |
| Child 1: yes/no Child 2: yes/no Child 3: yes/no Child 4: yes/no |
| Did you provide paracetamol to the child? |
| Child 1: yes/no Child 2: yes/no Child 3: yes/no Child 4: yes/no |
|  |
| **Paracetamol** |
| How many children received paracetamol before childcare attendance? Number: |
